# Changes in brain structure and function during early aging in patients with chronic low back pain

**DOI:** 10.3389/fnagi.2024.1356507

**Published:** 2024-06-07

**Authors:** Yao Zu, Zhou Zhang, Zengming Hao, Zimu Jiang, Ke Chen, Yu Wang, Changcheng Zou, Le Ge, Qiuhua Yu, Fuming Zheng, Chuhuai Wang

**Affiliations:** ^1^Department of Rehabilitation Medicine, the First Affiliated Hospital, Sun Yat-sen University, Guangzhou, China; ^2^College of Rehabilitation Medicine, Fujian University of Traditional Chinese Medicine, Fuzhou, China; ^3^College of Rehabilitation Medicine, Gannan Medical University, Ganzhou, China

**Keywords:** chronic low back pain, early aging, cognition-related brain regions, brain structure, brain function, fMRI

## Abstract

**Objective:**

To explore the structural and functional changes in cognition-related brain regions in patients with chronic low back pain (CLBP) at earlier ages, and explore the impact of the interaction between CLBP and age on the brain.

**Methods:**

Seventy-six patients with CLBP were recruited and divided into “younger” age group (20–29 years, YA), “middle” age group (30–39 years, MA), and “older” age group (40–49 years, OA). All patients underwent functional magnetic resonance imaging (fMRI) as well as clinical psychological and pain-related symptoms assessments.

**Results:**

Structural analysis showed that patients in OA group had lower gray matter (GM) volumes in the orbitofrontal cortex (OFC) bilaterally and the right superior frontal gyrus (SFG) compared to YA group. The resting-state brain activity analysis showed that amplitude of low-frequency fluctuation (ALFF) values in the bilateral postcentral gyrus and left ventral medial prefrontal cortex (mPFC) were significantly different in the OA group. The functional connectivity (FC) in the right ventral dorsolateral prefrontal cortex (DLPFC) and the right insula was significantly decreased in the OA group compared to the YA and MA groups. Likewise, the FC in the left caudal parahippocampal gyrus (PHG) and left inferior parietal lobule (IPL) were significantly lower in the MA and OA groups compared to the YA group. In addition, both the structural properties and the FC values of these brain regions were significantly correlated with age.

**Conclusion:**

This preliminary study concludes that CLBP affects the aging process. The synergistic effects of CLBP and aging accelerate the functional and structural decline of certain areas of the brain, which not only affects pain processing, but are also may be associated with cognitive declines.

## 1 Introduction

The high prevalence of chronic pain and cognitive decline is a serious public health problem in the aging population (van Kooten et al., 2017; Blanton et al., 2023). Studies have shown that patients with chronic pain have a faster rate of cognitive decline than their peers, and patients with dementia, the most common neurodegenerative disease, have 50% odds of comorbid chronic pain (van Kooten et al., 2016). Therefore, chronic pain and cognitive function are strongly associated.

Recent studies in healthy adults have shown that cognitive decline becomes apparent from the fifth decade of life (De Luca et al., 2003; Borella et al., 2008). Studies have also identified considerable alterations in cognition-related brain structure and function among healthy individuals over the age of 50 years, and many of these brain regions are involved in pain processing (Martucci et al., 2014). Neuroimaging studies have found that functional and structural changes in the prefrontal cortex, cingulate cortex, hippocampus, and parahippocampal gyrus (PHG) are particularly pronounced in patients experiencing chronic pain (Tinnirello et al., 2021; Hung et al., 2022). Moreover, significant reductions in gray matter (GM) volume in brain regions such as the anterior cingulate cortex, orbitofrontal cortex (OFC), and dorsolateral prefrontal cortex (DLPFC) have been found in patients with chronic low back pain (CLBP) (Apkarian et al., 2004). Because cognitive decline is more severe in patients with chronic pain than in normal aging and because these brain regions are involved not only in pain processing but also in a wide range of cognitive-related functions, including attention, memory, and executive function, some studies have suggested that these alterations in brain regions may be associated with accelerated cognitive decline in patients with chronic pain (Oosterman and Veldhuijzen, 2016; Whitlock et al., 2017). However, because of the high prevalence of chronic pain in middle-aged and older adults (Woolf and Pfleger, 2003), most of the participants in studies exploring cognition and chronic pain were in this age group, and little is known about brain structure and function changes at earlier ages (e.g., younger than 40).

Although a few studies have found significant GM, brain function, and cognitive performance changes in relatively young individuals with chronic pain (under 50 years of age), the results have been inconsistent across studies (Jensen et al., 2013; Bagarinao et al., 2014; Chong et al., 2014; Ung et al., 2014; Labus et al., 2015). Based on the available research, it appears that significant changes in cognitive performance may not be observed among adults with chronic pain. Only some studies on migraines and fibromyalgia have reported significant reduction in executive function, visuospatial ability, processing speed, and memory function in these patients (Veldhuijzen et al., 2012; Le Pira et al., 2014). Several studies have shown that changes in gray and white matter can occur in different brain regions, including the prefrontal lobe, cingulate gyrus, insular lobe, thalamus, temporal lobe, hippocampus, precuneus, and subcortical regions. These changes have been observed in individuals with various conditions, such as Crohn’s disease, chronic pelvic pain, irritable bowel syndrome (IBS), and chronic low back pain (Agostini et al., 2013; Bagarinao et al., 2014; Labus et al., 2014; Ung et al., 2014). The incidental report of cognitive decline, together with more frequent reports of reduced gray and white matter integrity at a younger age, supports the idea that chronic pain is characterized by neurocognitive deficits already at this age, and brain plasticity may compensate for these deficits (Oosterman and Veldhuijzen, 2016). However, the wide age range in patients in these studies does not allow identification of the specific age at which the alterations begin, which is important for understanding the neural mechanisms underlying the accelerated cognitive decline associated with chronic pain.

In addition, chronic pain is associated with activity in multiple networks in the central nervous system, including sensory, emotional, and cognitive. The prefrontal region, anterior cingulate cortex, amygdala, ventral tegmental area, and nucleus accumbens are associated with affective aspects of pain and regulate emotional and motivational responses (Yang and Chang, 2019). Since these brain regions do not operate separately, functional and structural changes and interactions can affect emotional and cognitive aspects (Malfliet et al., 2017). Depression symptom is a frequent emotional disturbance experienced by these patients. The relationship between pain and depression is thought to be bidirectional, and the underlying neurobiology is “shared” between the two conditions. Patients with chronic pain and depression symptom tend to exhibit lower levels of monoamine neurotransmitters, including 5-hydroxytryptamine, dopamine, and norepinephrine. Changes in GM volume, functional connectivity (FC), and neural activation in the prefrontal lobe, hippocampus, and amygdala have been observed (Zheng et al., 2022). Moreover, similar changes in the brain structure and function have been found in other emotional disturbances associated with pain, such as anxiety and pain catastrophizing (Malfliet et al., 2017). Therefore, the role of psychological factors in chronic pain patients should be considered.

Considering that the vast majority of previous studies have investigated changes in the brain structure and function as well as cognitive performance in the older population, and the few studies conducted in the young population included wide age range, the specific age at which the changes begin is difficult to identify. Therefore, this study aimed to examine different age groups before the age of 50 to provide specific evidence for understanding the neural mechanisms underlying accelerated cognitive decline associated with chronic pain. The main objectives of this study were: (1) to explore whether these brain regions in patients with chronic pain undergo structural and functional changes at earlier ages, and (2) explore the impact of the interaction between CLBP and age on the brain. We hypothesized that chronic pain induces the premature aging of brain function and affects cognitive function with age.

## 2 Materials and methods

This was a cross-sectional observational study investigating chronic pain across different ages. The study protocol was approved by the Institutional Ethics Committee for Clinical Research and Animal Trials of the First Affiliated Hospital of Sun Yat-sen University ([2021]079), and the clinical registration number is ChiCTR2100042810.

In this study, patients with CLBP were included and divided into three groups according to age: the “younger” age group (20–29 years, YA), the “middle” age group (30–39 years, MA), and the “older” age group (40–49 years, OA). Resting-state functional magnetic resonance imaging (fMRI) was used to explore changes in brain function among patients at different ages, and the 3D-T1 anatomical image was used to explore structural changes. The numerical rating scale (NRS) was used to assess pain intensity, while the Self-Rating Depression Scale (SDS) and Self-Rating Anxiety Scale (SAS) were used to measure patients’ psychological status.

### 2.1 Participants

A total of 76 CLBP patients were recruited from the First Affiliated Hospital of Sun Yat-sen University. The inclusion criteria were as follows: (1) clinical diagnosis of CLBP with persistent pain >3 months; (2) aged between 20 and 49 years; (3) NRS score of pain intensity ≥3 points; (4) right-hand dominance; (5) no fMRI contraindication; (6) ability to complete the SDS and SAS scales; and (7) no other central nervous system diseases. Exclusion criteria were (1) patients with a clear “reg flag sign,” such as unilateral leg pain, intermittent claudication, unexplained weight loss, or nocturnal pain; (2) patients with a history of spinal surgery, fracture, dislocation, or other spinal diseases; (3) patients with concurrent severe central or peripheral nervous system disease; (4) patients with contraindications to magnetic resonance scanning or those who could not cooperate to complete the examination. Finally, 26 patients aged 20–29 years, 27 aged 30–39, and 23 aged 40–49 were recruited. All patients provided their written informed consents before participating. We offered a detailed explanation of the experiment’s purpose, procedures, possible risks, and emergency treatment measures to all participants. The effective size was calculated to be 0.42 in accordance with the pre-experiment DLPFC ALFF values as the primary outcome. To produce the power of 80% at an alpha level of 0.05, two tails, the sample size was 60. Considering that the potential for 10% data quality control in each group, the optimal total sample size was 66, and the sample size in this study was nearly at this level.

### 2.2 Data acquisition

Images were acquired with a Philips 3.0 T Ingenia MRI scanner with a 32-channel brain coil and scanned using standard radiofrequency head oil. Structural data were collected using a high-resolution 3D T1-weighted sequence with the following parameters: matrix size, 256 × 256; in-plane resolution, 1 mm × 1 mm; repetition time (TR), 7.2 ms; echo time (TE), 3.3 ms; flip angle, 7; and field of view (FOV), 256 mm × 256 mm, 176 slices, and no slice gaps. Resting state fMRI was conducted using a T2-weighted, single-shot, gradient-recalled planar imaging sequence, with the following parameters: matrix size, 64 × 63; in-plane resolution, 3.5 mm × 3.5 mm; TR, 2,000 ms; TE, 30 ms; flip angle, 90; FOV, 224 mm × 224 mm; and slice thickness, 3.5 mm. The number of slices was 33 and slice gap 0.7 mm; the number of signals averaged was 1. The scan sequence was random, and the scan area covered the entire brain. The total number of volumes was 240. Before the scanning, patients were instructed to lie on their backs, close their eyes, and remain still, especially with their heads. We made sure that the patients were familiar with the procedure. One experimenter adjusted the patients’ posture, and another one monitored the equipment outside. Finally, a total of 76 patients completed scanning.

### 2.3 Measures

We collected data on the basic characteristics of the patients, including age, sex, pain intensity, and duration. The SDS and SAS were used to evaluate patient psychological states. The SDS contains 20 items that evaluate the state of depression, with each item having 4 levels. The state of depression was evaluated as follows: normal, <50; mild depression, 50–59; moderate depression, 60–69; and severe, >70. The SAS is a self-reported scale, with 20 items that cover a variety of anxiety symptoms, including both psychological and somatic symptoms. Participants provided responses on a 4-point scale ranging from 1 to 4, with total scores ranging from 20 to 80. Participants completed these surveys independently.

### 2.4 Data processing

#### 2.4.1 fMRI data processing

The fMRI data processing was based on SPM12 software.^1^ The procedure included converting the data formats, scrapping the first 10 volumes, correcting slice timing, realigning, coregistration (this included aligning the 3D-T1 structural image of each subject to the average functional image of all subjects, registering the aligned 3D-T1 structural images to the standard functional image by the segmentation step, and finally matching the individual functional images to the registered structural images), spatially normalizing, performing nuisance covariates regression (the global signals, white matter signals, and cerebrospinal fluid signal were included as covariates), smoothing, linearly detrending, and filtering (0.01–0.08 Hz). In the present study, we included the DLPFC (Bakkour et al., 2013; Staffaroni et al., 2019) and mPFC (Caetano et al., 2012; Chong et al., 2023) as regions of interest (ROIs), considering the overlap between pain and cognition in brain regions. In addition, the hippocampus as well as the PHG (Pantel et al., 2003; Burke et al., 2018) were also included as ROIs in this study owing to their involvement in aging. The Human Brainnetome Atlas was used to extract these ROIs as mask (Fan et al., 2016). FC analysis between ROIs and the whole brain and Fisher’s transformation were performed separately. To ensure fMRI data signal continuity, we did not conduct remediation (e.g., “scrubbing, despiking”). After applying a multiple comparison correction based on Gaussian random field theory (voxel *p* values < 0.001, cluster *p* values < 0.05, two-tailed, to form clusters, the neighborhood size was six), we included the FC results of the ROIs in the entire brain in the subsequent analysis. The amplitude of ALFF methodology was then used to explore changes in the spontaneous activity of brain functions in the resting state in all subjects. Demographic indicators with significant differences between groups were included as covariates in the fMRI processing.

#### 2.4.2 Structural data processing

Structural MRI data were analyzed using the FreeSurfer version 6.0 software package,^2^ and the standard pre-processing steps included head movement correction, removal of cranial as well as non-brain tissue, tissue normalization, structural segmentation of GM, white matter, and subcortical nuclei, and mapping of gray matter volume of brain (Fischl et al., 1999). Brain regions were divided using aparc atlas, and each ROI was made into a standard spatial label, then these labels in the standard space were mapped onto each subject’s individual space using the mri_label2label function, and finally the GM volume value of each brain region, as well as the total intracranial volume (eTIV), were extracted from each subject’s individual space using the asegstats2table function. Subsequently, we explored differences in GM volume among chronic low back pain patients at different ages, and included eTIV as a covariate in subsequent statistical analyses.

### 2.5 Statistical analysis

Multivariate analysis of variance (MANOVA) was used to assess group differences in demographic data using SPSS version 24. The Shapiro–Wilk test was used to assess the normality of the data distribution. The Kruskal–Wallis test was used to compare non-normally distributed data. All statistical analyses were two-tailed. Statistical significance was set as *p* < 0.05. We employed Pearson correlation analyses to determine the correlation between neuroimaging data and age. Spearman’s correlation analyses were used to evaluate the non-normally distributed data. Outliers were eliminated using the boxplot method. Since sex, pain intensity, pain duration, SDS scores, or SAS spatially normalizing scores between the groups, they were not included as covariates in the correlation analysis. All results were adjusted through false discovery rate correction.

## 3 Results

### 3.1 Demographic and clinical characteristics

The demographic and clinical data of the participants are presented in Table 1. Among the groups, there were no significant differences in sex ratio, pain intensity, pain duration, SDS scores, or SAS scores.

**Table 1:** Demographic and clinical characteristics of the participants.

	YA 20–29 *N* = 26	MA 30–39 *N* = 27	OA 40–49 *N* = 23	Test statistics	*p* value
**Demographic features**					
Age, years	25.26 ± 2.62	35.07 ± 2.84	44.17 ± 2.85	285.127*	0.000
Sex ratio, male	9 (34.6%)	8 (29.6%)	8 (34.8%)	0.202**	0.904
**Pain symptom severity**					
Pain intensity, by NRS	5 (2)	5 (2)	5 (2)	0.404**	0.817
Pain duration, months	24 (24)	36 (65.50)	20 (96)	0.441**	0.802
SDS scores	49.24 ± 10.92	46.67 ± 14.39	49.46 ± 12.71	0.383*	0.683
SAS scores	43.46 ± 8.66	45.23 ± 11.31	45.87 ± 10.43	0.374*	0.689

*ANOVA test. ** Kruskal-Wallis test. The results satisfying normal distribution were expressed as means ± standard deviations, otherwise were expressed as median (inter-quartile range). NRS, numerical rating scale; SDS, Self-Rating Depression Scale; SAS, Self-Rating Anxiety Scale.

### 3.2 Neuroimaging results

**Figure 1:**
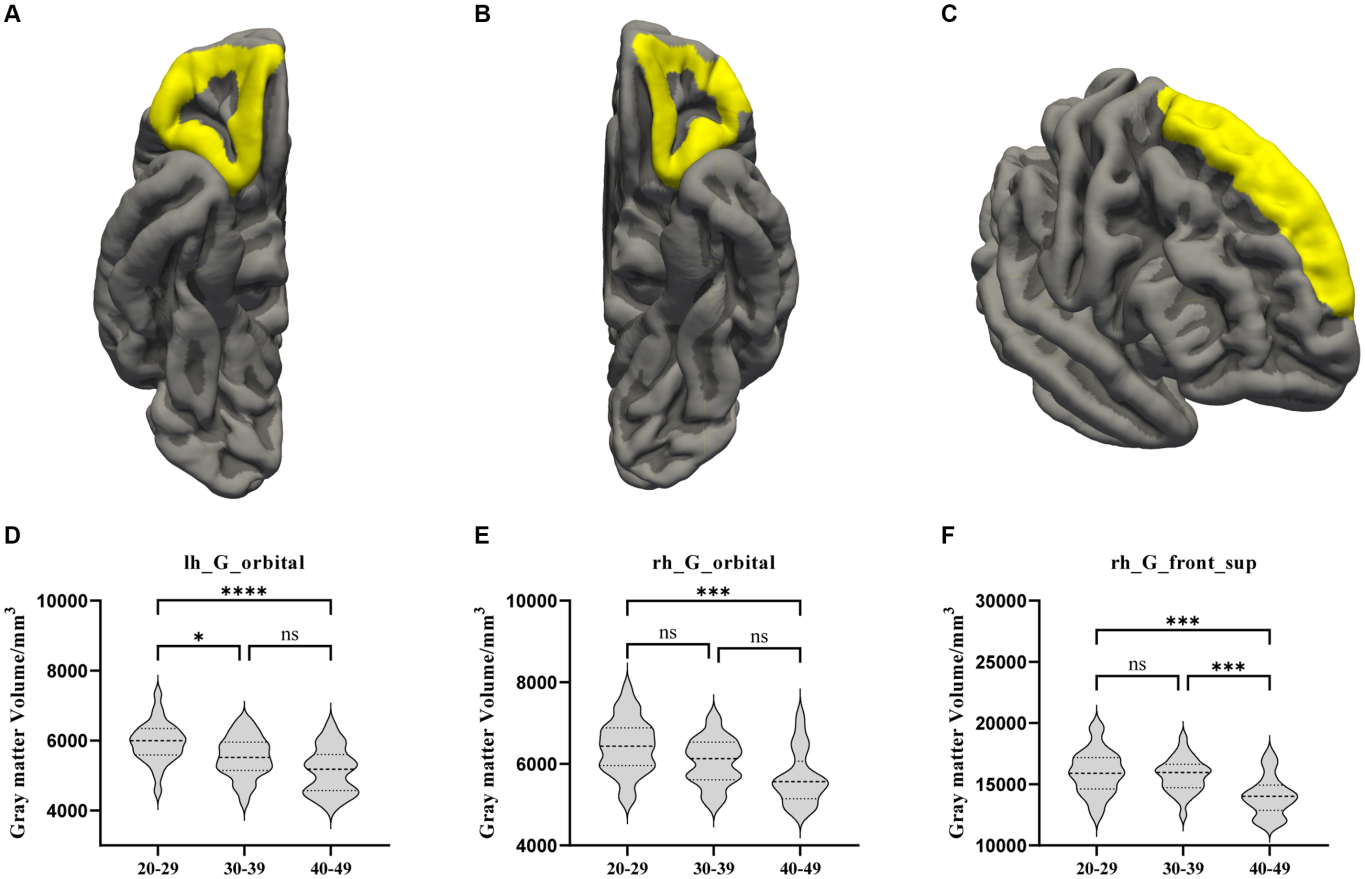
The GM volume results with significant difference among the YA, MA, and OA groups. **(A)** Location of the left OFC, **(B)** Location of the right OFC, **(C)** Location of the right hemisphere. **(D–F)** Showed the brain regions of GM volume differences in three groups. All results were threshold at *p* < 0.05 (FDR corrected). *p < 0.05, **p < 0.01, ***p < 0.001, ****p < 0.0001. ns, not significant; GM, gray matter; OFC, orbitofrontal cortex; SFG, superior frontal gyrus.

Comparing the whole brain GM volume in the three groups, the GM volumes in both the OFC bilaterally and the right SFG were significantly lower in the OA group than in the YA group. There was also a significant difference in left OFC volume between the YA and MA groups. In the right SFG, the GM volume was lower in the OA group than in MA group (Figure 1).

**Figure 2:**
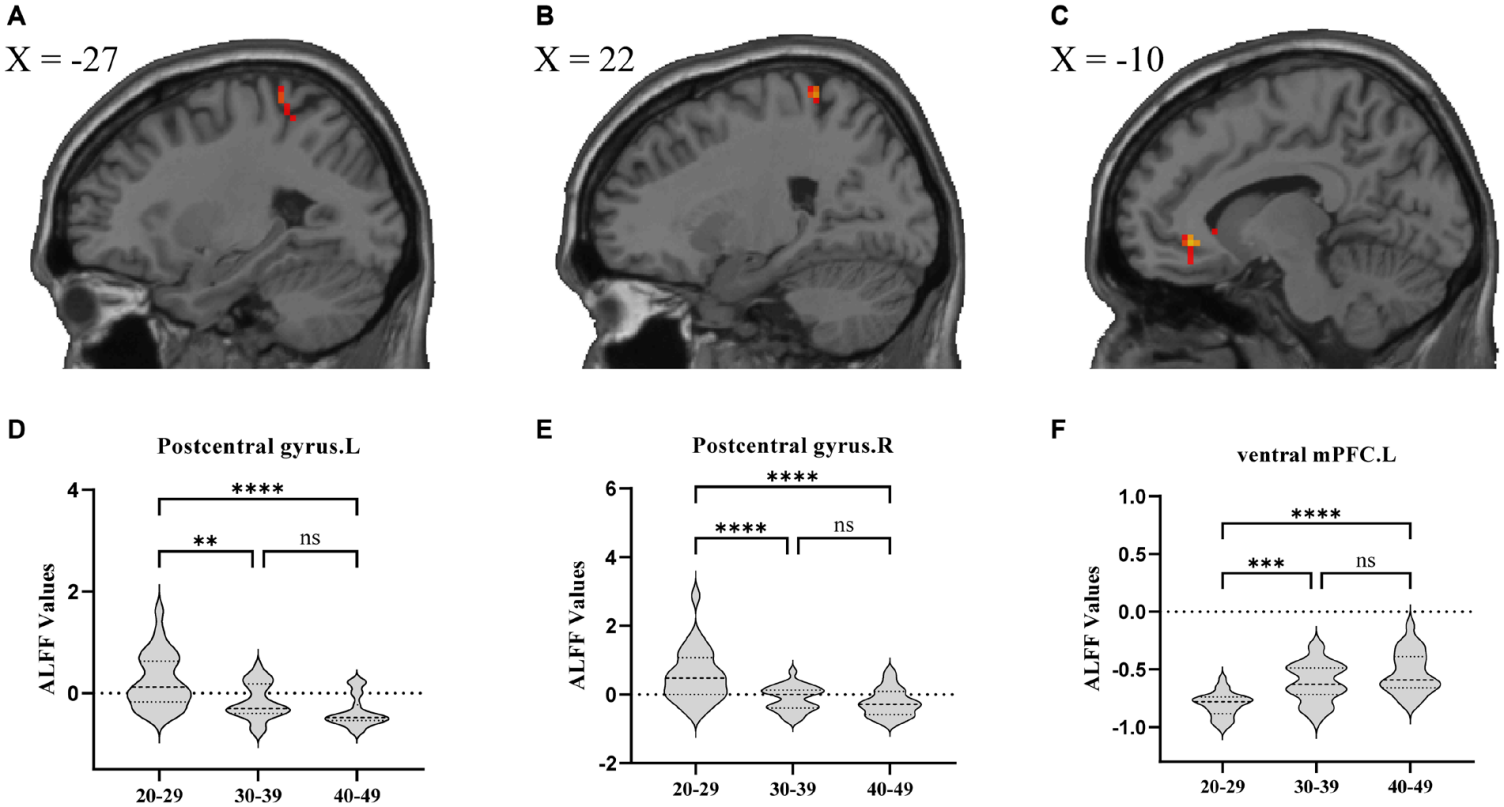
ALFF analysis comparing patients among the YA, MA, and OA groups. **(A)** Location of left postcentral gyrus, **(B)** Location of right postcentral gyrus, **(C)** Location of left ventral mPFC. **(D–F)** Showed the brain regions of ALFF differences in three groups. All results were threshold at *p* < 0.05 (FDR corrected). *p < 0.05, **p < 0.01, ***p < 0.001, ****p < 0.0001. ns, not significant; ALFF, amplitude of low frequency fluctuation; mPFC, medial prefrontal cortex.

Regarding brain region activity, compared to the YA group, amplitude of ALFF values in the bilateral postcentral gyrus were significantly decreased in the MA and OA groups. However, ALFF value in the left ventral mPFC were significantly increased in the MA and OA groups. In addition, ALFF values were not significantly different between the MA and OA groups (Table 2; Figure 2).

**Table 2:** The brain regions with significant FC and ALFF between groups.

Regions	R/L	Cluster size voxels	MNI (Peak)			Peak intensity
			*x*	*y*	*z*	
Right ventral DLPFC-INS	R	25	42	−3	3	12.0316
Left caudal PHG-IPL	L	64	−48	−48	51	12.3746
Ventral mPFC	L	27	−12	36	−6	17.7496
Postcentral gyrus	L	19	−27	−39	69	10.4367
Postcentral gyrus	R	15	24	−42	69	14.4559

FC, functional connectivity; ALFF, amplitude of low frequency fluctuation; MNI, Montreal Neurological Institute; R/L, right or left; DLPFC, dorsolateral prefrontal cortex; INS, insula; PHG, parahippocampal gyrus; IPL, inferior parietal lobule; mPFC, medial prefrontal cortex.

**Figure 3:**
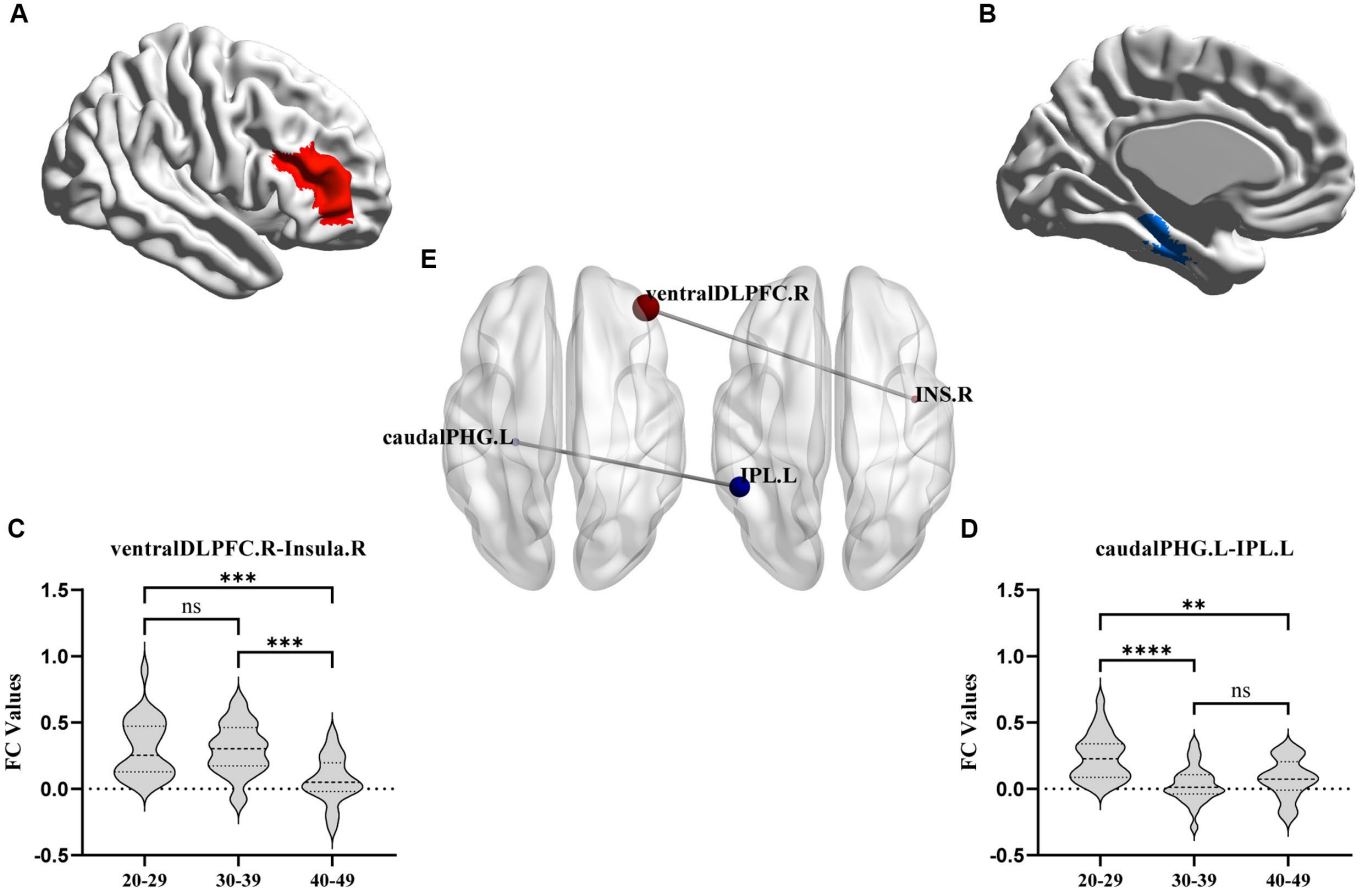
FC results with significant difference among YA, MA, and OA groups. **(A,B)** axial view. **(C,D)** Showed the difference of connectivity among three groups from ROI-ROI analysis. The parameters represent the FC correlation coefficient. *Represents the FC of brain regions with significant correlation after FDR correction. **p < 0.01, ***p < 0.001, ****p < 0.0001. FC, functional connectivity; DLPFC, dorsolateral prefrontal cortex; PGH, parahippocampal gyrus; IPL, inferior parietal lobule.

FC analysis showed that, compared to the YA and MA groups, the FC between the right ventral DLPFC and the right insula was decreased significantly in the OA group. The FC between the left caudal PHG and the left IPL in the MA and OA groups was significantly lower than that in the YA group (Table 2; Figure 3).

**Figure 4:**
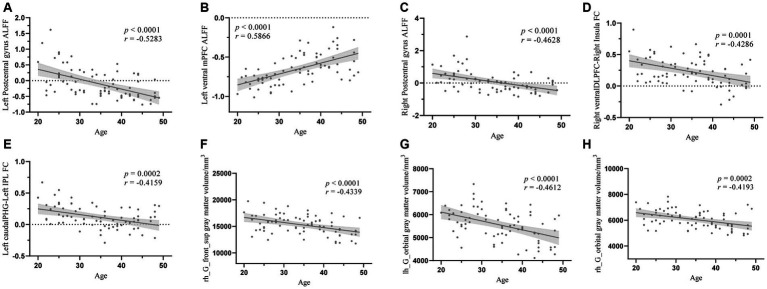
The correlation analysis between age and structural properties and the FC values. **(A)** The correlation analysis between age and left postcentral gyrus ALFF, **(B)** The correlation analysis between age and left ventral mPFC ALFF, **(C)** The correlation analysis between age and right postcentral gyrus ALFF, **(D)** The correlation analysis between age and right ventralDLPFC-right insula FC, **(E)** The correlation analysis between age and left caudalPHG-left IPL FC, **(F)** The correlation analysis between age and right SFG gray matter volume, **(G)** The correlation analysis between age and left OFC gray matter volume, **(H)** The correlation analysis between age and right OFC gray mater volume. ALFF, amplitude of low frequency fluctuation; mPFC, medial prefrontal cortex; DLPFC, dorsolateral prefrontal cortex; FC, functional connectivity; PHG, parahippocampal gyrus; IPL, inferior parietal lobule.

Correlation analysis showed that age was significantly negatively correlated with left postcentral gyrus ALFF (*r* = −0.5283, *p* < 0.0001), right postcentral gyrus ALFF (*r* = −0.4628, *p* < 0.0001), right ventral DLFC-right insula connectivity (*r* = −0.4286, *p* = 0.0001), left caudal PHG-left IPL connectivity (*r* = −0.4159, *p* = 0.0002), right hemisphere SFG GM volume (*r* = −0.4339, *p* < 0.0001), left hemisphere OFC GM volume (*r* = −0.4612, *p* < 0.0001), and right hemisphere OFC GM volume (*r* = −0.4193, *p* = 0.0002). Furthermore, age was significantly positively correlated with the ALFF of the left ventral mPFC (*r* = 0.5866, *p* < 0.0001). All the results were adjusted through false discovery rate correction (Figure 4).

## 4 Discussion

This study investigated the structural and functional changes and their coupling in cognition-related brain regions in patients with CLBP before the age of 50 years. We divided the patients into different age groups to offer new insights into the impact of chronic pain on early aging. We present three major findings: (1) compared to the YA group, there was a statistically significant decrease in the right SFG and bilateral OFC GM volumes in chronic pain patients aged 40–49 years, and the difference became more pronounced with increasing age; (2) compared to the YA group, the ALFF in the left ventral mPFC of CLBP patients was increased significantly in OA and MA, and the activity of the left mPFC was positively correlated with age; (3) compared to the YA group, CLBP patients aged between 40 and 49 years had significantly decreased FC of the right ventral DLPFC and the right insula, as well as reduced FC of the left caudal PHG and the left IPL. Moreover, FC values showed a gradual decline with increasing age. These results suggested that CLBP seems to accelerate the functional and structural decline in some of the brain regions, and this acceleration becomes significant in a certain age group.

This study investigated alterations in the brains of patients in specific age groups and examined the differences between age groups to elucidate the influence of CLBP on cognition-related brain regions relatively early in life. After comparing the thicknesses and volumes of whole brain regions among the three age groups, we found an overall trend of decreasing GM volume among all groups, especially the GM volumes of the bilateral OFC and right SFG in the OA group. Moreover, correlation analysis conducted between the SFG and OFC GM volumes and age revealed a significant negative correlation. This finding suggests that patients with CLBP under the age of 50 years experienced significant structural changes in the right SFG and bilateral OFC at the age of 30–39 years, and these changes were progressively increased over time. Previous studies in other chronic pain patients under 50 years of age, such as those with Crohn’s disease (Agostini et al., 2013), fibromyalgia (Jensen et al., 2013), IBS (Seminowicz et al., 2010; Labus et al., 2014), and headaches or migraines (Messina et al., 2013; Magon et al., 2015), have found similar results showing reduced GM volume in the SFG. Similarly, a decrease in GM volume in the OFC has been observed in certain cases of chronic tension-type headache (Schmidt-Wilcke et al., 2005) and IBS (Labus et al., 2015). However, these studies included a wide range of ages, and our study delved deeper into ascertaining the age at which changes occurred in this particular brain region. In addition to the alterations observed in chronic pain, the SFG and OFC that undergo relatively marked atrophy in normal aging have been highlighted (Raz and Rodrigue, 2006; Bakkour et al., 2013). The SFG plays an essential role in memory and higher-order cognitive processing, particularly in working memory (Briggs et al., 2020). The OFC is a core brain region correlated with episodic memory (Rolls et al., 2022; Tang et al., 2022; Wang et al., 2023). These brain alterations were found to expand with age in the present study, suggesting that chronic pain may lead to earlier functional and structural decline in the OFC and SFG, thereby potentially impairing pain processing and memory function. Another possible memory-related finding in this study was that the FC between the left caudal PHG and the left IPL became significant at the age of 30–39 years and decreased considerably with age. Previous studies on normal aging have found that GM volume decline in the PHG and IPL is significantly associated with memory (Huo et al., 2018); fMRI studies on Alzheimer’s disease have similarly found that the patients experienced significant reduction in FC between the PHG and IPL (Qi et al., 2018). These results confirmed that CLBP may accelerate memory loss.

In the ALFF analyses, we found that, compared with the YA group, both the MA and OA groups demonstrated a significant increase in left ventral mPFC ALFF values. Although there were no significant differences between the MA and OA groups, correlation analysis identified a significant positive association between the mPFC ALFF values and age. Hence, it was suggested that the activity in the left ventral mPFC was increased significantly in patients with CLBP aged 30–39 years, and that this change became increasingly noticeable with age. Previous studies of chronic back pain as well as studies of subacute back pain have found similar results to those in the present study (Hashmi et al., 2013). The mPFC not only plays an important role in ascending and descending pain control but also acts as a crucial region in negative emotion, reward processing, and memory (Yu et al., 2014; Iwabuchi et al., 2015; Meints et al., 2019; Tran et al., 2023). Considering that the presence of negative emotions, such as depression symptoms and apathy, increases with age in the normal older population (McCue, 1995; Gunning et al., 2021), we speculated that the decline in emotional control may be more severe in patients with CLBP than in the normal older population.

Our study also found that, compared to the YA group, there was a significant decrease in ALFF values in the bilateral postcentral cortex in the MA and OA groups. However, no significant differences were observed between the MA and OA groups. Additionally, correlation analysis revealed a significant negative correlation between the ALFF values in the bilateral postcentral cortex and age. These results suggest that in patients experiencing CLBP under 50 years of age, the bilateral postcentral gyrus ALFF values were significantly altered at the age of 30–39 years, and the alterations continued to increase with age. A number of previous studies have similarly observed significant changes in ALFF values for the primary somatosensory area (S1) in patients with CLBP (Zhang et al., 2019; Tong et al., 2023); these studies either had a wide age range (20–50 years) or were limited to a specific age group, which did not allow to evaluate trends in these changes and the time points at which they occurred. The S1 plays an important role in pain localization, distinguishing pain intensity, and modulating neuronal activity resulting from both harmful and harmless stimuli in downstream areas of the brain linked to pain transmission (Kim et al., 2017). In normal aging, sensitivity to pain perception decreases at 60 years of age and continues to decline over time (Lautenbacher, 2012; Lautenbacher et al., 2017). However, considering that the time point at which S1 alterations were found to occur were much earlier compared to normal aging in the present study, whether this suggests that sensory localization, differentiation of pain intensity, and other sensory-related functions decline prematurely in patients with CLBP warrants further in-depth investigation.

Some of the FC analysis results are worth discussing. The changes in FC between the ventral DLPFC and the right insula were significant in those aged 40–49 years, and these changes increased with age. Given the important role of the DLPFC and insula in pain perception (Lorenz et al., 2003; Labrakakis, 2023), it is easy to conclude that this function begins to decline in patients with CLBP in this age range. Similarly, previous studies have identified the important role of DLPFC-insula FC in pain tolerance (Lautenbacher et al., 2017; Geisler et al., 2021). Therefore, the early modulation of these two brain regions in young patients experiencing pain would be beneficial for pain management to avoid a decline in these two important functions. In addition, in aging studies, it has been found that the strength of FC between the DLPFC and insula was reduced in healthy older adults and that this connectivity was strongly associated with a decline in decision making ability (Čeko et al., 2015; Weissberger et al., 2020); thus, whether the presence of reduced FC between the DLPFC and insula in younger individuals in the present study implies an earlier onset of decision-making cognitive decline in patients with CLBP likewise deserves further exploration.

Our study had several limitations. Firstly, our study only recruited 20–49 years old CLBP patients, age-and gender-matched healthy control groups were absent. However, few studies have investigated the effect of chronic pain on early aging, and our findings provide some clues for further study. Secondly, there was an absence of true aging groups with and without CLBP. Our results were limited to the current age groups. Further studies should recruit a wider age range of CLBP patients and healthy controls in order to fully evaluate the changing trajectories of brain structure and function. Thirdly, the cognitive performances of patients were not evaluated. During the discussion, the alterations in the brain structure and function, along with the potential modifications in cognitive function, were speculated based on previous studies. In addition, the factors affecting cognitive performance include not only the brain regions, but also the level of education, living lifestyle, and psychological status. Thus, we cannot draw definite conclusions based on the brain regions alone. Considering these limitations, future studies should include specific cognitive tests to assess patients’ cognitive abilities.

## 5 Conclusion

This preliminary study concluded that CLBP affects the aging process. The synergistic effects of CLBP and aging accelerate the functional and structural decline of certain areas of the brain, which not only affect pain processing but are also possibly associated with cognitive decline, including memory, emotional regulation, and decision-making. Moreover, we anticipate that our results will raise awareness among younger patients and emphasize the importance of preventing cognitive decline.

## Data availability statement

The raw data supporting the conclusions of this article will be made available by the authors, without undue reservation.

## Ethics statement

The studies involving humans were approved by Institutional Ethics Committee for Clinical Research and Animal Trials of the First Affiliated Hospital of Sun Yat-sen University. The studies were conducted in accordance with the local legislation and institutional requirements. The participants provided their written informed consent to participate in this study.

## Author contributions

YZ: Conceptualization, Investigation, Methodology, Resources, Writing – original draft. ZZ: Data curation, Formal analysis, Investigation, Methodology, Writing – original draft. ZH: Formal analysis, Methodology, Resources, Writing – review & editing. ZJ: Data curation, Formal analysis, Methodology, Resources, Writing – review & editing. KC: Investigation, Writing – review & editing. YW: Investigation, Writing – review & editing. CZ: Investigation, Writing – review & editing. LG: Resources, Writing – review & editing. QY: Data curation, Funding acquisition, Writing – review & editing. FZ: Methodology, Resources, Supervision, Writing – review & editing. CW: Conceptualization, Funding acquisition, Methodology, Supervision, Writing – review & editing.

## Footnotes

^1^http://www.fil.ion.ucl.ac.uk/spm/

^2^https://surfer.nmr.mgh.harvard.edu
